# Assessment of clinical outcomes and histomorphometric findings in alveolar ridge augmentation procedures with allogeneic bone block grafts: A systematic review and meta-analysis

**DOI:** 10.4317/medoral.23353

**Published:** 2020-02-10

**Authors:** Fabián Pérez-González, Pedro Molinero-Mourelle, Luis Sánchez-Labrador, Luis Miguel Sáez-Alcaide, Alvaro Limones, Jorge Cortés-Bretón Brinkmann, Juan López-Quiles

**Affiliations:** 1 Department of Dental Clinical Specialties, Faculty of Dentistry, Complutense University of Madrid, Spain; 2 Department of Conservative Dentistry and Orofacial Prosthodontics, Faculty of Dentistry. Complutense University of Madrid, Spain

## Abstract

**Background:**

This systematic literature review aimed to evaluate the efficacy of allogeneic bone blocks for ridge augmentation by assessing block survival rates and subsequent implant survival, including post-surgical complications and histomorphometric analysis.

**Material and Methods:**

An electronic and manual search among references, was conducted up to April 2019 by two independent authors. Inclusion criteria were: human clinical trials in which the outcomes of allogeneic bone block grafts were evaluated by means of their survival rates and subsequent implant success rates.

**Results:**

Seven articles fulfilled the inclusion criteria and were analyzed. A total of 323 allogeneic block grafts were monitored for a minimum of 12 months follow-up after surgery, of which thirteen (4.02%) failed. Regarding the cumulative implant survival rate, the weighted mean was 97.36%, computed from 501 implants. Histologic and histomorphometric analysis showed that allogeneic block grafts presented some clinical and microstructural differences in comparison with autologous block grafts.

**Conclusions:**

Atrophic alveolar crest reconstruction with allogeneic bone block grafts would appear a feasible alternative to autologous bone block grafts, obtaining a low block graft failure rate, similar implant survival rate and fewer postoperative complications. Further investigations generating long term data are needed to confirm these findings.

** Key words:**Allogeneic block, clinical outcome, ridge augmentation, dental implants.

## Introduction

As the world population ages and dental loss increases, implant dentistry is subject to growing demands and new challenges. Following tooth loosening due to periodontal disease, caries, trauma, or tumoral processes, bone resorption and remodeling of the alveolar ridge makes the insertion of implants difficult (1,2). In cases of atrophic maxilla or mandible, several treatment options are available that make implant placement possible such as guided bone regeneration, split crest technique, sinus lift, osteogenic distraction, or bone blocking, among others (3,4).

The use of autologous bone, which can be combined with a membrane, is considered by some authors the gold standard for bone regeneration due to its osteogenic, osteoinductive and osteoconductive properties (2,3,5,6). Autologous bone can be harvested from different regions including intraoral and extraoral sites. Nevertheless, these grafts provide limited quantities of bone, and are associated with morbidity, in particular neurosensory disturbance at intraoral harvesting sites (1,3,6-8).

Allogeneic bone grafts used to treat intraoral defects were first described by Narang *et al*. (9). Allogeneic bone blocks offer a viable alternative to autologous bone, as they can be obtained in unlimited quantities from a human tissue bank, are of limited antigenicity, and present a low risk of disease transmission thanks to a complex process of delipidization, oxidation, dehydration, and gamma irradiation (1,10,11).

Although, the allogeneic bone block graft technique is simpler, this technique, does not present identical behavior to autologous grafts and to date scientific evidence in support of its use is relatively scarce (11,12).

Due the lack of studies and standardized protocols, with firmly inclusion criteria and medium- and long-term follow-up, we deem justified to carry out a systematic review. In turn, the objectives of this systematic review concern participants, interventions and outcome should respond following statement of questions: what are the survival rates of dental implants placed in allogeneic bone blocks? What are the blocks survival rates and what is the bone resorption rate of allogeneic bone blocks? Which surgical complications may occur? and which histologic and histomorphometric findings do we observe?.

Materials and Methods

- Protocol development and PICO question

The review protocol was developed to meet PRISMA (Preferred Reporting Items for Systematic Review and Meta-Analyses) guidelines (Moher, Liberati, Tetzlaff, & Altman) (13), designed to answer the following PICO (Population, Intervention, Comparison, Outcome).

However, the established focus question was an adaptation to a PIO question: “In edentulous and partially edentulous patients (P), what are the survival rates, complication rates, and histologic findings following surgery (O) of allograft blocks and implants placed in augmented sites (I)?”, taking into account that comparison (C) is not applicable because it did not exist a control group.

- Eligibility criteria 

Population: Systemically healthy edentulous and partially edentulous patients.

Intervention: Allograft block grafting procedures to increase the alveolar ridge at dental implant sites.

Comparison: Not applicable.

Outcomes: Survival rates and complication rates of allograft blocks and implants placed in augmented sites; bone gain and bone resorption of bone blocks, histological and histomorphometric analysis of graft sites.

- Inclusion criteria

1. Clinical studies reporting data on: survival rates; technical, biologic and aesthetic complication rates of dental implants placed in allogeneic bone block grafted areas; bone blocks survival rates, histological and histomorphometric findings.

2. Follow-up of at least one year.

3. Human studies with a sample size greater than five.

4. Randomized controlled clinical trials, cohort studies, case-control studies, cross-sectional studies, and case series were included.

5. English and Spanish language and with no-time restrictions.

- Exclusion criteria

1. Studies with pooled results that did not allow a distinction between the results.

- Type of intervention and comparisons

Studies were selected that included interventions for treating atrophic maxillae and/or mandibles by means of allogeneic bone block grafts.

- Outcomes

The primary outcome used to assess the management of atrophic maxillae and mandibles was the survival rates of allogeneic bone blocks and subsequently the implants placed in augmented sites.

The following secondary outcomes were studied: the intra- and post-operative complications of both allogeneic bone blocks and implants, bone gain and resorption rate in allogeneic bone blocks, changes in marginal bone levels and the histologic and histomorphometric findings.

- Search strategy

An electronic search for studies published up to April 2019 was conducted in four databases: 1. The National Library of Medicine (MEDLINE via PubMed); 2. Cochrane Central Register of Controlled Trials; 3. SCOPUS; and Web of Science (WOS).

For Pubmed and Scopus databases, the search strategy was: 1# (“Allogeneic bone block graft” OR “Bone block graft”) AND (“Dental implants” OR “Dental implantation, Endosseous”), in advance mode with no filters.

In addition, a manual search was conducted in dentistry and implantology scientific journals for articles published in English and Spanish until April 2019.

- Screening methods

Two reviewers (FPG and PMM) screened the titles and abstracts of articles in the electronic and manual searches independently. Then, full texts of the studies selected, where screening of those which abstract supplied insufficient information to reach a decision. Any disagreement was resolved by discussion with a third reviewer (JCBB).

- Data extraction

The same two reviewers performed data extraction in duplicate. When data was incomplete or missing, the authors of the study were contacted for clarification. When the results of a study were published more than once, only the longest follow-up was included.

- Quality assessment (risk of bias in individual studies)

The Newcastle-Ottawa scale (NOS) for cohort studies and a modification of the scale for cross-sectional studies were used to assess risk of bias in individual observational studies and non-randomized trials (Wells *et al*., 2011) (14).

- Statistical Analysis

The survival rate of implants and allogeneic block grafts was calculated analyzing the failure events among the total implants and block grafts placed in the included studies with a confidence interval (CI) of 95% using fixed or random-effect models depending on the heterogeneity of the included trials, and it was represented through forest plot. The Cochran’s Q test and I^2 were used to determine the statistical heterogeneity. If the I^2 value is between 0 and 50% and *p-value* of the Q test is > 0.05, the level of heterogeneity was interpreted to be within accepTable limits, and therefore, a fixed-effect model would be applied. The implant survival rate placed in allogeneic block grafts was calculated with the same methodology mentioned above. The analyses were performed using Comprehensive Meta-Analysis version 3 software (Biostat, Englewood, NJ, USA) by A.L.

## Results

- Study selection

**Figure 1:**
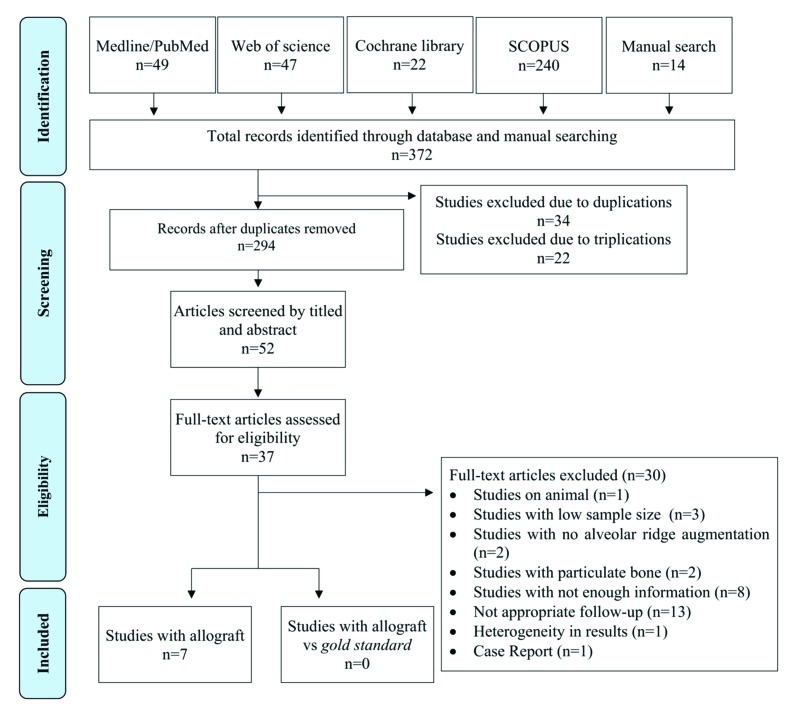
Flow chart of the inclusion and exclusion of studies in this review.

The initial electronic database search located 358 articles and the manual search a further 14 articles. Of the total 372 articles, 66 were duplicates and were discarded. After an initial screening to eliminate articles not relevant to the PIO question, followed by title and abstract screening, the full texts of a total of 52 articles were read. After reading the full text, a total of seven studies fulfilled the inclusion criteria and were selected for data extraction and analysis. A flow diagram (Fig. 1) illustrates the entire search and selection process.

- Study characteristics

Information about the selected studies including study design, study objectives, sample size, assessment methodology, follow-up period, and other data are shown in Table 1 and Table 2.

**Table 1:**
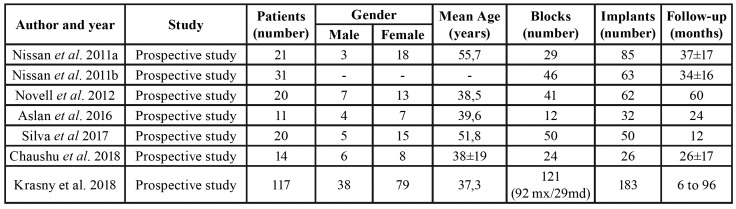
Description of included studies.

**Table 2:**
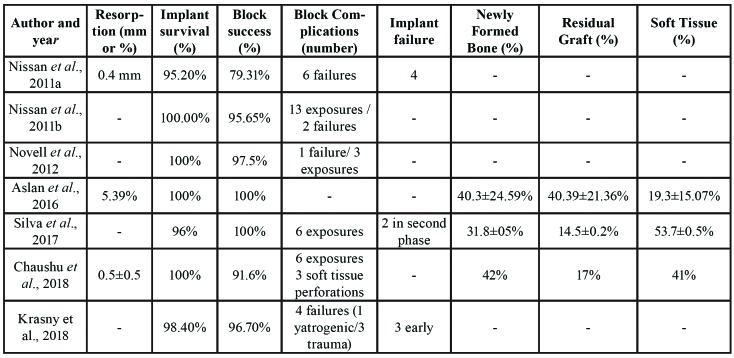
Description of clinical outcomes and histological findings.

- Risk of bias

Quality assessment of the case control and cohort studies reviewed is summarized in Table 3. According to the NOS scale, one study scored 2 points, two 3 points, and four obtained 4 points.

**Table 3:**
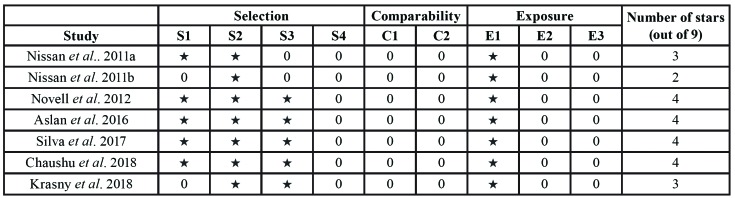
Quality assessment of included studies using the Newcastle-Ottawa scale.

- Synthesis of results

Inter-investigator agreement

Inter-reviewer reliability of full-text analysis was calculated (percentage of agreement and kappa correlation coefficient). Calculated K-range was 0.955 (CI 95%).

It had been decided to divide the studies into two groups: studies that used allogeneic bone blocks alone, and studies that compared allogeneic bone blocks with autologous bone blocks. However, the latter group failed to fulfill inclusion criteria due to insufficient follow-up times and presented very heterogeneous results, making comparison difficult.

- Patient characteristics

A total of 234 patients (n=234) were recruited in allogeneic bone block studies. Patient ages ranged from 18 to 80 years, and the sample included 63 men and 140 women; only one study did not report patient age or gender.

Regarding the classification of maxillary and mandibular defects, there was homogeneity between the studies. The main requirement was an alveolar ridge between of 3-5 mm.

- Block survival rate

**Figure 2:**
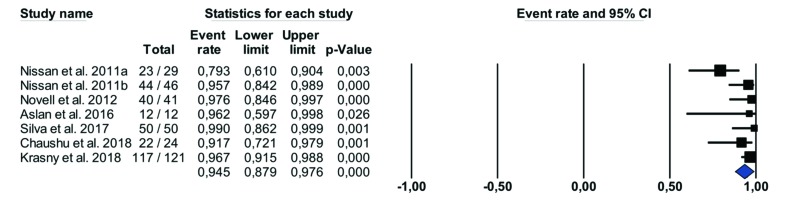
Survival rates of allogenic block graft.

A total of 323 allogeneic bone blocks were grafted, but most studies did not provide information about location; only one work with 121 allogeneic bone blocks stated that 92 were grafted in the maxilla and 29 in the mandible (15). Only one study showed a survival rate lower than 90% (79.31%) (4). When the survival rate of allogeneic block grafts was calculated, statistical heterogeneity was detected (Cochran`s Q (df = 6) = 13.726, *p* (value) = 0.033, I^2 = 56.286%). Therefore, a random-effects model was chosen. The meta-analysis results show that the overall survival rate of allogeneic block grafts was 94.52 %, Fig. 2. Both allogeneic bone blocks and implants were monitored for a minimum 12-month follow-up. The longest follow-up was of 96 months (15).

- Implant survival rate 

**Figure 3:**
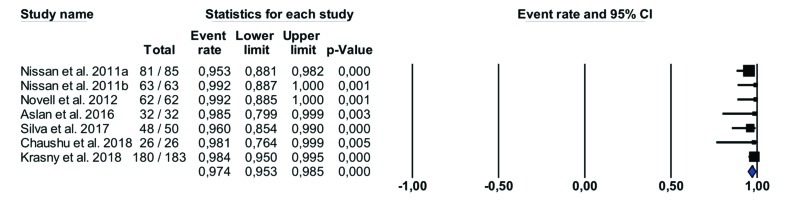
Survival rate of implants placed in allogeneic block graft.

A total of 501 dental implants were placed and the survival rates were reported in all studies, ranging between 95.2 % and 100%. When the implant survival rate placed in allogeneic block grafts was calculated, statistical heterogeneity was not detected (Cochran`s Q (df = 6) = 4.143, *p* (value) = 0.657, I^2 = 00.00%). Therefore, a fixed-effects model was chosen. The meta-analysis results show that the overall survival rate of the implants was 97.36%, Fig. 3. The implant healing period varied from 3 to 6 months.

- Bone gain

Only three studies of allogeneic bone block grafts included information about horizontal or vertical bone gain, horizontal gain ranging between 1.65±0.14mm (12) and 4.69mm (15), while vertical bone gain depended on the healing time, from 5.15 mm after surgery to 2.92mm after a one-year follow-up (15,16).

- Block resorption

The results for block resorption were heterogeneous due to the different types of data provided. Two studies report resorption in millimeters, ranging between 0.4 and 0.5±0.5mm (4,17), while another study measured resorption of the original bone block as a percentage, with a mean of 5.39±2.18% (12).

- Marginal bone loss

Only one article measured and reported data on marginal bone loss around implants where the amount was 0.5±0.5mm after a follow-up period of 37±17 months (4).

- Treatment complications: blocks and implants

Regarding bone block complications, six studies reported postoperative complications: twenty-eight allogeneic bone blocks suffered exposure and nine osseointegration failure (4,10,15-18). One study reported four failed bone blocks, three of them due to prosthetic trauma and the other due to iatrogenic dislocation (15).

Regarding implant complications, four studies reported postoperative complications: eleven implants failed due to lack of osseointegration, and three were lost within the first month after placement (15).

- Histologic and histomorphometric findings

Newly formed bone, residual bone graft, and soft tissue were evaluated in only four of the articles reviewed. In three works concerning allogeneic bone blocks, newly formed bone ranged between 31% and 42% on average; residual bone graft varied from 14% to 40%, while marrow and connective tissue ranged between 19.3 and 53.7% (12,16,17).

## Discussion

This systematic review analyzed allogeneic block grafts used for bone reconstruction procedures in atrophic maxillae and mandibles for subsequent dental implant placement. It also analyzed the complications and histomorphometric findings reported in the studies reviewed.

Although autologous bone, harvested from intraoral and extraoral sites, is described by some authors as the ‘gold standard’ graft material for bone regeneration due to its osteogenic, osteoinductive and osteoconductive properties, it also suffers several disadvantages such as higher morbidity; the need for a donor site, surgery, and extended hospitalization; and its limited availability (19).

The most common intraoral donor sites are the mandibular ramus and the chin. These areas provide adequate availability, and the resorption rates reported range between 6.9% and 18% (20-22). In fact, the study by Gultekin *et al*. is noTable because the autologous bone block resorption rate was found to be lower (7.2±1.4%) than with guided bone regeneration (GBR) (12.48±2.67%), while Chappuis *et al*., with a 10-year follow-up, found no considerable increase in bone block resorption rate.

The main disadvantages of intraoral autologous bone block grafting are the complications derived from the surgical technique that some authors report as occurring in 30-50% of cases. Of these, the most serious is neurosensory disturbance (19,23,24). Neurosensory disturbances are seen to appear most often in autologous chin bone blocks (19,23,24), which can also produce some aesthetic changes in the patient´s facial contour (23).

In this context, the use of allogeneic bone blocks would appear a viable option that offers several advantages: shorter surgery time, unlimited availability, lower morbidity and no risk of neurosensory disturbances (12,25). In terms of osseointegration, allogeneic bone blocks have been reported to show successful host bone responses thanks to their osteoinductive potential (26). Moreover, the allogeneic bone block makes it possible to plan ahead and customize the graft material, adapting it to the bone defect to be regenerated (25).

The mean allogeneic block osseointegration rate of the 347 blocks included in the present literature review was 94.5% (Fig. 2), which is similar to autologous bone block rates (27). Furthermore, the resorption rate of the blocks was very low, 5.39±2.18% according to Aslan *et al*. and 0.4-0.5 ± 0.5mm according to Chaushu *et al*. However, in the studies reviewed, there was no standardized protocol for quantifying resorption and a high proportion of the studies did not investigate this outcome. Nevertheless, Lumetti *et al*. reported that, for fresh-frozen allogeneic bone block grafts, the resorption rate was higher than that of autologous bone (46% vs. 28%), although denser allogeneic bone blocks may offer a more accepTable resorption rate (28).

For the 501 dental implants placed in the studies reviewed, the survival rate in grafted allogeneic bone block areas is 97,4% (Fig. 3) which is similar to survival rates for conventional implant placement or implants inserted in areas grafted with autologous bone. According to Papaspyridakos *et al*. (29), marginal bone loss around implants, is expected to be a maximum of 1.5mm during the first year but in the studies reviewed marginal bone loss was not evaluated with the exception of two works: Nissan *et al*. 2011 (4) obtained 0.5± 0.5mm after 37±17 months follow-up, while Park *et al*. (30) compared marginal bone loss with allogeneic blocks (0.38mm) and autologous bone blocks (0.15mm) finding no statistically significant differences between the grafts.

Unlike the complications associated with autologous bone blocks, with allogeneic bone blocks neurosensory disturbances rarely occur. The most common complication in the studies analyzed was block exposure, described in 30 blocks (4.32% of all allogeneic bone blocks), reported by Nissan *et al*. (18) and Chaushu *et al*. (17), in which approximately 30% of the allogeneic bone block suffered exposure or soft tissue dehiscence.

Regarding the histology, the autologous bone blocks presented perfect integration and often the interphase was difficult to identify, and the quantity of necrotic bone was minimal. On the other hand, the interphase in allogeneic bone block grafts showed a clearly differentiated necrotic area (31). But no statistically significant differences in newly vital bone formation were reported. It is worth noting that a significant difference was found between allogeneic and autologous residual graft (28.9% allogeneic vs. 19.5% autologous) (32).

The studies included found percentages of newly formed bone ranging from 31% to 42%, residual bone graft of between 14% and 40%; and soft tissue or empty areas varied between 19.3% and 53.7% (12,16,17).

Studies comparing allogeneic with autologous bone blocks obtained better results with autologous bone in terms of newly vital bone, but the difference between the two types of graft did not reach statistical significance (32). Moreover, the study by Lorenz *et al*. described the presence of multinucleated giant cells in biopsies. These cells normally appeared in the presence of a foreign body cell. Nevertheless, the amount was minimal (0.82 ± 2.97 MNCG/mm2) (33).

Although it was found that resorption, survival rates and complications associated with allogeneic bone blocks were accepTable, there was a lack of standardization in the studies: some authors waited four months (34), while others waited six or more months before implant placement (25,35). One author fixed the block with a fixation screw (10) while another used titanium plates (32). Some studies used resorbable membranes and particulate bone (16) while others did not used any biomaterial (3). There was no consensus as to the best implant design: tapered or straight (17).

In spite of the accepTable result in terms of osseointegration of both blocks and implants, bone gain, survival rate and associated complications in allogeneic bone block, there is a lack of quality studies comparing allogeneic versus autologous bone block in order to stablish adequate conclusions.

## Conclusions

According to the findings of this systematic review, it may be concluded that allogeneic bone blocks are an adequate option for regeneration of atrophic maxilla and mandible – avoiding the need for autologous bone block harvest surgery – in terms of survival rates of bone blocks and subsequent implants. However, there is a lack of standardization in the literature, so more studies are necessary with correct protocols, adequate sample sizes and follow-up periods, which would provide clear and reliable results.
